# A Novel Case of Biliary Stent Migration Causing Sigmoid Diverticula Entrapment

**DOI:** 10.7759/cureus.39322

**Published:** 2023-05-22

**Authors:** Anmol Mittal, Afif Hossain, Kamal Amer, Ayham Khrais, Siddharth Verma

**Affiliations:** 1 Department of Medicine, Rutgers New Jersey Medical School, Rutgers University, Newark, USA; 2 Department of Gastroenterology and Hepatology, Rutgers New Jersey Medical School, Rutgers University, Newark, USA; 3 Department of Gastroenterology and Hepatology, East Orange Veterans Affairs Medical Center, East Orange, USA

**Keywords:** plastic biliary stent, follow-up appointment, acute gi bleed, biliary diseases, biliary stent migration

## Abstract

Pancreaticobiliary obstruction is a rare but life-threatening complication. Plastic biliary stents are a temporary utility to maintain the patency of the common bile ducts, typically lasting about four months. Biliary stents can rarely have complications, with the most common being migration through the gastrointestinal tract. We present a case of a patient with a plastic stent placed over five years, which was complicated by severe hematochezia due to the retention of the stent in a diverticulum. Given the increased risk of life-threatening complications post-stent life expectancy, there should be systems in place to prevent patients from being lost to follow-up.

## Introduction

In the United States, over 20 million Americans are affected by gallbladder disease [1]. Of those with gallbladder disease, 1% to 4% will develop symptomatic gallbladder disease, with 4% of the general population developing choledocholithiasis [2,3]. Although pancreaticobiliary obstruction is a rare complication of gallstone disease, it can be life-threatening. Accordingly, biliary stenting with plastic prostheses has become the gold standard in the setting of biliary ductal obstruction. The patency of biliary stents is highly variable, largely dependent on the type of stent placed and the indication for stenting. In general, self-expanding metal stents (SEMSs) are patent for a longer duration than plastic stents (PS), with covered stents being patent for longer than uncovered stents. Patency for PS is usually less than four months and between six and 12 months for SEMS. Generally, there is a higher risk for complications with PS compared to SEMS but no difference in mortality at one month [4]. As such, most PS are removed or exchanged within one to three months, and most SEMS within three to six months. Common complications of biliary stenting include migration, pancreatitis, and obstruction of the biliary stent (stone vs. sludge), leading to cholangitis [5]. Commonly, stents placed can become dislodged and pass through the gastrointestinal (GI) tract spontaneously. Once they traverse the ileocecal valve, most stents pass spontaneously. Here, we report a unique case of lower GI bleeding secondary to plastic biliary stent dislodgement within a diverticulum five years after placement. 

This article was previously presented as a meeting abstract at the 2020 American College of Gastroenterology (ACG) Annual Scientific Meeting on October 25, 2021.

## Case presentation

**Figure 1:**
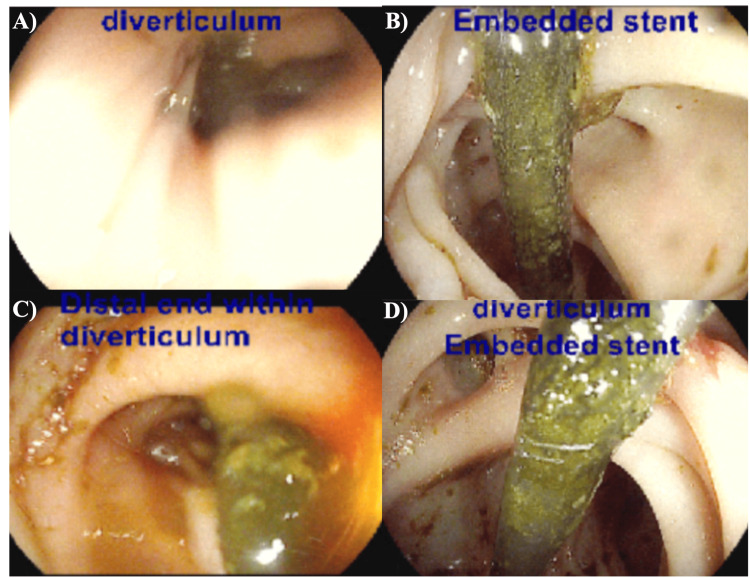
(A) Sigmoid diverticulum; (B) plastic biliary stent visualized adherent to colonic mucosa and embedded within sigmoid diverticulum; several distal diverticula can be visualized as well; (C) direct luminal view within sigmoid colon along the axis of plastic biliary stent embedded within diverticula; and (D) another view of plastic biliary stent following removal from the sigmoid diverticulum; fresh blood clots can be visualized in the surrounding diverticula.

A 72-year-old veteran (male) with a known history of hypertension, peripheral vascular disease, cholecystitis status after laparoscopic cholecystectomy complicated by biliary leak requiring the placement of two biliary stents, prior polysubstance use, and post-traumatic stress disorder presented with a two-month history of functional decline and weight loss failure to thrive. His labs were significant for the following: WBC 10.3, aspartate transaminase (AST)/alanine transaminase (ALT) 153/94, alkaline phosphatase (ALP) 703, total bilirubin 1.5, procalcitonin 2.24, C-reactive protein (CRP) 190.3, erythrocyte sedimentation rate (ESR) 64, gamma-glutamyl transpeptidase (γ-GTP) 609, fractionated alkaline phosphatase 87% liver isozyme, and CA 19-9 of 494. The patient was subsequently admitted for further management, given the concern for malignancy. He was started on broad-spectrum antibiotics and intravenous fluid resuscitation; however, he developed three episodes of hematochezia with a drop in hemoglobin from 14.6 to 9.0 with associated symptoms. He underwent a colonoscopy, which revealed a dislodged stent with its pigtail entrapped within a sigmoid diverticulum. Bleeding stigmata were visualized adjacent to the stent (Figures 1A-1D). The stent was removed via rat-tooth endoscopic forceps, allowing for control of bleeding and resolution of the patient’s hematochezia. 

## Discussion

There are 40 published cases involving foreign bodies and sigmoid diverticular disease [6]. To our knowledge, only 10 of these cases presented with biliary stent migration causing sigmoid diverticula entrapment or perforation. The persistence of a biliary stent for greater than three to six months significantly increases the risk of complication, especially with PS. The risk of postoperative stent migration is estimated between 5% and 10% and increases depending on the time the stent is in place. PS migrate more frequently than SEMS (10% compared to 1%, respectively) [7]. Fortunately, most displaced stents pass spontaneously, and those that have not passed are successfully retrieved via endoscopic measures with greater than 90% efficacy [5]. Only a minority of cases require surgical intervention. Accordingly, it is extremely uncommon for a stent to remain for five years. 

We speculate this patient had recent stent dislodgement, leading to common bile duct occlusion and resultant cholangitis. After localization of the biliary stent in the sigmoid colon on CT, the patient was initially managed conservatively for spontaneous passage through the ileocecal valve. However, once the patient developed a hemodynamically significant GI bleed, endoscopic retrieval of the displaced stent became paramount. After endoscopic stent retrieval, intravenous fluids, and blood transfusion, the patient became hemodynamically stable. This case underscores the importance of creating a robust standardized post-stenting follow-up schedule. As time elapses post-stenting, the risk and severity of complication increase and can have deleterious consequences. Thus, every effort should be made to avoid loss of follow-up to prevent life-threatening complications of biliary stent complications. This can be accomplished by creating an internal hospital registry where each stent placed has a follow-up assigned to ensure the removal of the stent. This may allow for the prevention of adverse outcomes like those present in this patient. 

## Conclusions

Patients with pancreaticobiliary obstructions are at high risk for morbidity and mortality. The gold standard in treatment is the placement of temporary stents to bypass the blockage and provide a conduit for easy bile flow. Stent migration can occur in a small percentage of patients with the risk increasing from the time since placement, and though most pass spontaneously, some do not. It is important to ensure that close follow-up is established with patients who receive this intervention as a rare but life-threatening gastrointestinal bleed, as in our patient, may occur.
